# Lipoprotein(a) in Vietnamese Cardiologists: Findings From a Pilot Study at the Vietnam Atherosclerosis Society Congress

**DOI:** 10.1002/hsr2.71436

**Published:** 2025-11-06

**Authors:** Thanh Huong Truong, Tien Anh Hoang, Van Sy Hoang, Ngoc Quang Nguyen, Hoa Tran, Duc Chinh Nguyen, Huu Duc Nguyen, Hai Nguyen Ngoc Dang, Thang Viet Luong, Doan Loi Do, Mai Ngoc Thi Nguyen, Hong An Le, Thanh Tung Le, Ngoc Thanh Kim, Quang Binh Truong

**Affiliations:** ^1^ Faculty of Medicine Phenikaa University Ha Noi Vietnam; ^2^ Department of Internal Medicine University of Medicine and Pharmacy, Hue University Hue Vietnam; ^3^ Department of Internal Medicine University of Medicine and Pharmacy at Ho Chi Minh City Ho Chi Minh City Vietnam; ^4^ Department of Cardiology Cho Ray Hospital Ho Chi Minh City Vietnam; ^5^ Department of Cardiology Hanoi Medical University Ha Noi Vietnam; ^6^ Vietnam National Heart Institute Bach Mai Hospital Ha Noi Vietnam; ^7^ Interventional Cardiology Department University Medical Center Ho Chi Minh City Vietnam; ^8^ Department of Cardiology Can Tho S.I.S General Hospital Can Tho Vietnam; ^9^ Department of Cardiology Quang Tri General Hospital Quang Tri Vietnam; ^10^ Faculty of Medicine Duy Tan University Da Nang Vietnam; ^11^ Menzies Institute for Medical Research University of Tasmania Hobart Tasmania Australia

**Keywords:** atherosclerotic cardiovascular disease, cardiovascular risk, lipoprotein(a), statin therapy, Vietnam

## Abstract

**Background and Aims:**

Lipoprotein(a) (Lp[a]) is an independent cardiovascular risk factor. Although current guidelines recommend Lp(a) testing, physicians are seldom screened, even though they remain at risk and often overlook their own health. In Vietnam, data on Lp(a) remain unclear. To address this, the Vietnam Atherosclerosis Society launched a pilot study to assess elevated Lp(a) among Vietnamese cardiologists, aiming to generate initial data, encourage physician screening, and raise medical and public awareness.

**Methods:**

A cross‐sectional study was conducted at the 2024 Vietnam Atherosclerosis Society Congress, inviting 800 cardiologists. After exclusions, 165 without cardiovascular disease were analyzed. Demographic, biochemical, and lipid profiles were collected, and Lp(a) was measured using the Tina‐quant Lp(a) Gen 2 assay.

**Results:**

Elevated Lp(a) levels (≥ 125 nmol/L) were observed in 12.12% of the participants. There were no significant differences in median age (*p* = 0.488) or sex distribution (*p* = 0.328) between participants with and without elevated Lp(a). Lp(a) levels were not correlated with other lipid parameters, body mass index, or age. No significant difference in Lp(a) levels was observed between statin users and nonstatin users. Among participants who achieved LDL‐C and non‐HDL‐C treatment targets, 8% still presented elevated Lp(a) levels.

**Conclusion:**

At the Vietnam Atherosclerosis Society Congress, elevated Lp(a) levels were detected in several cardiologists without prior cardiovascular disease, including those with well‐controlled lipid profiles according to current guideline targets.

## 1 Introduction

Atherosclerotic cardiovascular disease (ASCVD) is a major global health burden and was identified as the leading cause of morbidity and mortality in 2022, affecting over 500 million individuals and accounting for approximately 19 million deaths worldwide [1]. This leads to acute cardiovascular events such as myocardial infarction and ischemic stroke while also increasing the risk of chronic kidney disease and peripheral vascular disease [2].

Low‐density lipoprotein cholesterol (LDL‐C) is now recognized not only as a risk factor but also as a primary causal agent in the development of atherosclerosis [3]. Numerous clinical trials have demonstrated that lipid‐lowering therapies, particularly those that reduce LDL‐C, can significantly lower cardiovascular risk and provide substantial benefits for patients with ASCVD [4, 5]. In addition to LDL‐C, residual cardiovascular risk factors continue to contribute to ASCVD. However, even with effective LDL‐C control, residual cardiovascular risk factors persist and continue to contribute to ASCVD [6].

Among these factors, lipoprotein(a) (Lp[a]) has emerged as a critical yet often overlooked risk factor. Once considered part of the “hidden” residual risk, Lp(a) is now recognized as an independent, genetically determined risk factor for ASCVD [7, 8]. This discovery highlights the complexity of cardiovascular disease management and presents ongoing challenges for cardiologists in addressing the full spectrum of risk factors.

Furthermore, elevated Lp(a) levels are prevalent globally [9, 10], affecting approximately one in five individuals, highlighting its critical importance. Recognizing its impact, clinical guidelines have progressively emphasized the need for assessing and managing elevated Lp(a) to mitigate its associated cardiovascular risks [11, 12].

Despite these international recommendations, awareness and use of Lp(a) testing in Vietnam remain low. Within the healthcare system, Lp(a) is rarely included in routine practice, and many physicians are unaware of its importance or clinical implications. Furthermore, doctors themselves are seldom screened, even though they may have elevated cardiovascular risk and often neglect their own health [13].

This lack of local data makes it difficult to integrate Lp(a) into national strategies for lipid disorder management. To begin addressing this gap, we conducted a pilot study at the 2024 Vietnam Atherosclerosis Conference. The aim of this study was to explore the distribution of Lp(a) levels among Vietnamese physicians, especially cardiologists, who attended the conference. By generating preliminary evidence, this study provides a foundation for future research and supports the development of national clinical strategies for Lp(a) management. More importantly, it emphasizes the need to prioritize physician health as a key component of public health policy in Vietnam.

## 2 Methods

### 2.1 Study Design and Study Population

This cross‐sectional study was conducted at the 2nd Annual Scientific Congress of the Vietnam Atherosclerosis Society (VAS), held at Nha Trang City, from August 9–10, 2024. The study was approved by the Vietnam Atherosclerosis Society (approval number: 01/VAS‐NCKH) and adhered fully to the principles of the Declaration of Helsinki (2013 version). The reporting followed the STROBE guidelines to ensure the quality of observational research.

Previous studies have reported that the prevalence of elevated Lp(a) in Asia ranges from 10% to 25% [14, 15, 16]. Using the sample size calculation formula [17], we selected an acceptable margin of error of 10% and used the highest reported prevalence (25%) to estimate the largest required sample size. On the basis of these parameters, the minimum sample size needed was 72 cases [18].

**Figure 1:**
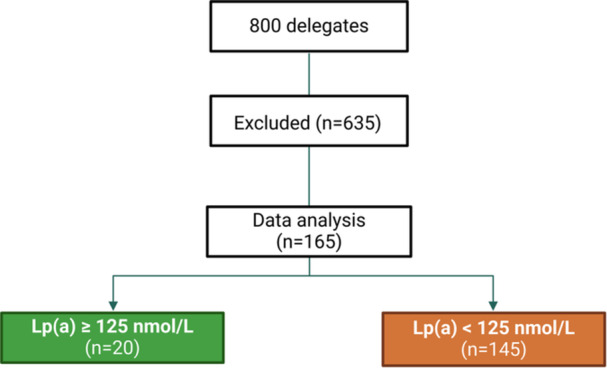
Flowchart of participant selection and data analysis. Abbreviation: Lp(a), lipoprotein(a).

In our study, we employed a convenience sampling method by screening 800 Vietnamese cardiologists who attended the 2024 Vietnam Atherosclerosis Society Congress. All participants were practicing physicians in Vietnam and were invited to take part in the study on a voluntary basis. Exclusion criteria included participants with a history of cardiovascular diseases, such as coronary artery disease, cerebrovascular disease, aortic disease, or peripheral arterial disease. Additionally, individuals with chronic kidney disease, chronic liver disease, or thyroid disorders were excluded. Those who declined to participate were also not included. After applying these criteria, we selected 165 participants for analysis. Details of the sample selection process are illustrated in Figure 1.

### 2.2 Patient and Public Involvement

Patients and/or the public were not involved in the design, conduct, reporting, or dissemination plans of this study.

### 2.3 Study Variables and Measurements

Demographic information of the participants, including age, sex, height (m), and weight (kg), was collected through a survey administered during the conference.

Height measurement: Participants stood upright in a natural posture with their head positioned so that the outer canthus of the eye and the external auditory meatus were aligned horizontally, parallel to the ground. The backs of the head, upper back, buttocks, and heels were in contact with the measuring scale. Height was measured from the ground to the highest point of the head and recorded in centimeters (cm) with an accuracy of 0.5 cm.

Weight measurement: The weighing scale was calibrated and placed on a flat surface before measurement, ensuring that the needle pointed to zero. The participants removed shoes, hats, and any items in their pockets or hands before stepping lightly onto the center of the scale. They stood upright, facing forward. Weight was recorded in kilograms (kg) with an accuracy of 0.5 kg.

Body mass index (BMI) was calculated via the following formula: BMI = weight (kg)/height² (m²), with units expressed in kg/m².

Biochemical blood tests were conducted on fasting venous blood samples. Lipid profiles, including total cholesterol, triglyceride, high density lipoprotein cholesterol (HDL‐C), and low density lipoprotein cholesterol (LDL‐C), were analyzed via enzymatic methods, with the results expressed in mmol/L.

Data on tobacco use, alcohol consumption, physical inactivity, comorbidities, and current medication use were obtained through structured self‐report questionnaires administered to all participants.

To assess lipid control, we applied the 2019 European Society of Cardiology/European Atherosclerosis Society Guidelines for the Management of Dyslipidaemias: Lipid Modification to Reduce Cardiovascular Risk. The recommended targets for LDL‐C and non‐HDL‐C were used to stratify risk levels. Specifically, non‐HDL‐C goals are set at < 2.2 mmol/L (< 85 mg/dL) for very‐high‐risk individuals, < 2.6 mmol/L (< 100 mg/dL) for high‐risk individuals, and < 3.4 mmol/L (< 130 mg/dL) for moderate‐risk individuals. Additionally, LDL‐C targets are < 1.4 mmol/L (< 55 mg/dL) for the very‐high‐risk group, < 1.8 mmol/L (< 70 mg/dL) for high‐risk individuals, < 2.6 mmol/L (< 100 mg/dL) for moderate‐risk individuals, and < 3.0 mmol/L (< 116 mg/dL) for low‐risk individuals [11].

### 2.4 Lipoprotein(a) Testing

Fasting venous blood samples were collected for testing and stored at 2°C–8°C. The samples were transported to the laboratory within 8 h after collection at the 2nd Vietnam Atherosclerosis Society Congress. The testing was conducted via Roche's Cobas analytical system. Lp(a) levels were measured with the Tina‐quant Lp(a) Gen 2 assay, which employs a particle‐enhanced turbidimetric immunoassay. The results are reported in nanomoles per liter (nmol/L). The analytical measurement range for the assay was 7–240 nmol/L, with a detection limit of 7 nmol/L. In this study, we used the American College of Cardiology and American Heart Association recommended threshold of ≥ 125 nmol/L to define elevated Lp(a) levels [19].

### 2.5 Statistical Analysis

Statistical analyses were performed via SPSS Version 26 (IBM, New York, United States) and GraphPad Prism Version 10 (GraphPad Software, Boston, United States). All the statistical tests were two‐sided, with the significance level set at *p* < 0.05. The statistical analysis followed the SAMPL guidelines to ensure clarity and accuracy in reporting [20]. Data normality was assessed via the Kolmogorov‒Smirnov test. Normally distributed continuous variables are presented as the means ± standard deviations, whereas nonnormally distributed variables are expressed as medians with interquartile ranges. Categorical variables are summarized as frequencies and percentages. Fisher's exact test was used to assess differences in categorical variables between groups. For continuous variables, either the unpaired *t* test or the Mann‒Whitney U test was used, depending on the data distribution. Missing data were excluded from all analyses. Spearman's correlation coefficient was applied for nonnormally distributed variables, and Pearson's correlation coefficient was used for normally distributed variables to determine correlations between continuous variables.

## 3 Results

The study was conducted with 165 cardiologists with no history of cardiovascular diseases at the Vietnam Atherosclerosis Society Congress 2024. This study revealed significant differences in lipid profiles between sexes. Triglyceride levels are significantly greater in males, whereas HDL‐C and Lp(a) levels are significantly greater in females (*p* < 0.001 and *p* = 0.037, respectively). Other parameters, including age, BMI, cholesterol, and LDL‐C levels, were not significantly different. Further details are provided in Table 1.

**Table 1:** Characteristics of the study population.

Characteristics	Total (*n* = 165)	Male (*n* = 60)	Female (*n* = 105)	*p* value
Age (years)	35.48 ± 11.54	35.13 ± 10.02	35.69 ± 12.37	0.755
Smoking (%)	5 (3.0)	5 (8.3)	0 (0.0)	**0.006**
Alcohol consumption (%)	26 (15.8)	15 (25.0)	11 (10.5)	**0.025**
Physical inactivity (%)	19 (11.5)	6 (10.0)	13 (12.4)	0.801
BMI (kg/m^2^)	22.28 ± 2.87	21.83 ± 2.95	22.55 ± 2.80	0.128
Cholesterol (mmol/L)	5.01 ± 1.12	5.20 ± 1.12	4.90 ± 1.11	0.100
Triglyceride (mmol/L)	1.54 [0.98–2.39]	2.25 [1.24–3.29]	1.27 [0.92–1.84]	**< 0.001**
HDL‐C (mmol/L)	1.36 [1.12–1.56]	1.25 [0.96–1.37]	1.45 [1.23–1.7]	**< 0.001**
LDL‐C (mmol/L)	3.03 [2.48–3.80]	3.04 [2.57–3.89]	3.0 [2.36–3.72]	0.416
Non‐HDL‐C (mmol/L)	3.51 [2.89–4.50]	3.40 [2.81–4.20]	3.77 [3.26–4.90]	0.293
Lipoprotein(a) (nmol/L)	15.65 [8.25–56.27]	12.7 [5.8–12.8]	18.4 [9.8–62.9]	**0.037**

*Note:* The values are presented as the means ± standard deviations, medians [IQRs], or *n* (%) as appropriate.

Abbreviations: BMI, body mass index; HDL‐C, high‐density lipoprotein cholesterol; LDL‐C, low‐density lipoprotein cholesterol.

**Figure 2:**
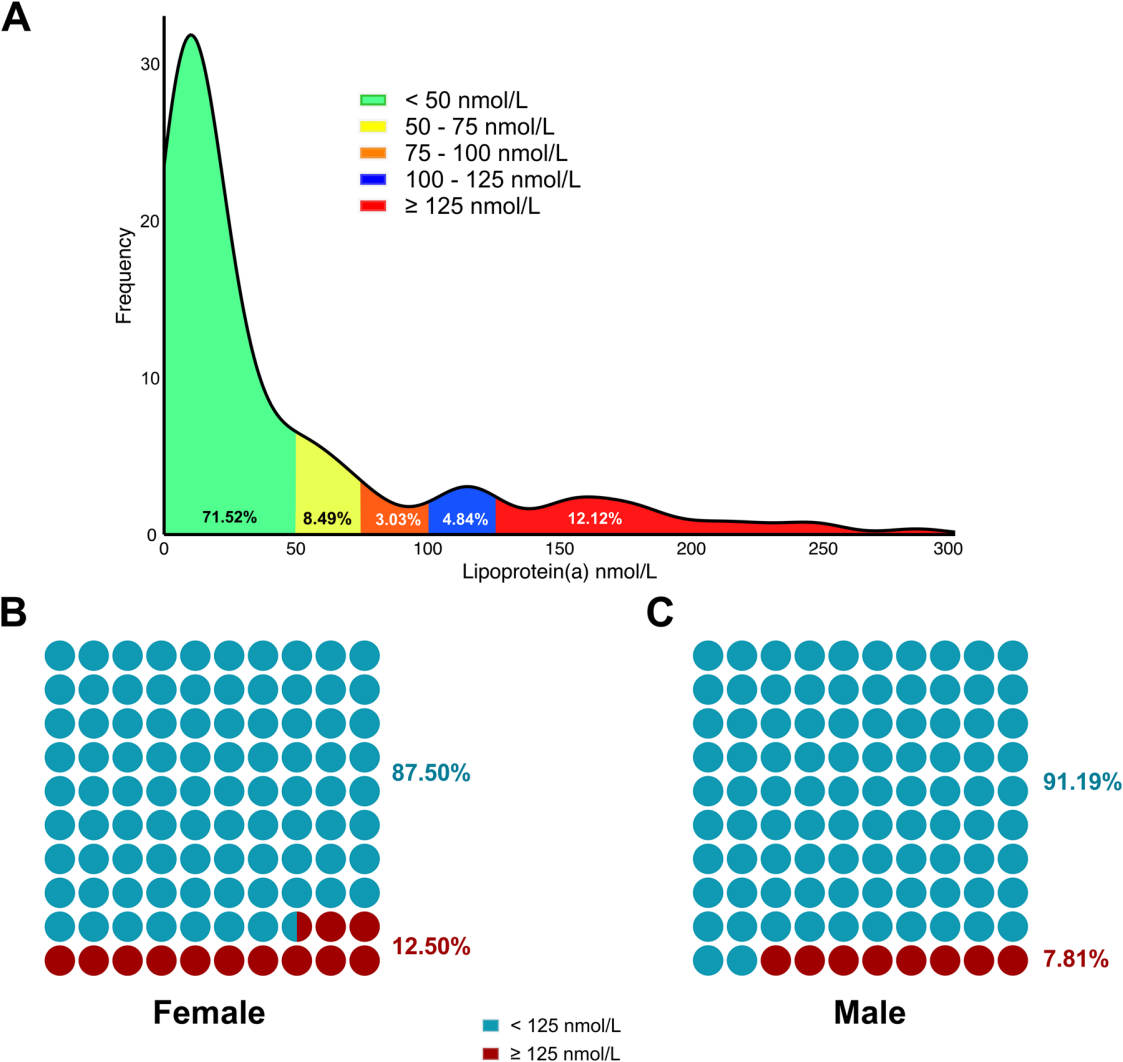
Distribution of Lp(a) levels among the study participants. (A) Histogram illustrating Lp(a) categories based on clinical thresholds; (B) Proportion of female participants with Lp(a) ≥ 125 nmol/L; and (C) Proportion of male participants with Lp(a) ≥ 125 nmol/L.

Figure 2 illustrates the distribution of Lp(a) levels among the study participants. Overall, 71.52% of the participants had Lp(a) levels less than 50 nmol/L, whereas 12.12% had levels ≥ 125 nmol/L, indicating that a notable proportion of the participants had elevated cardiovascular risk. Gender‐specific analysis revealed that 12.50% of females had Lp(a) levels ≥ 125 nmol/L, whereas 7.81% of males did, suggesting a slightly greater prevalence of elevated Lp(a) levels in females.

**Figure 3:**
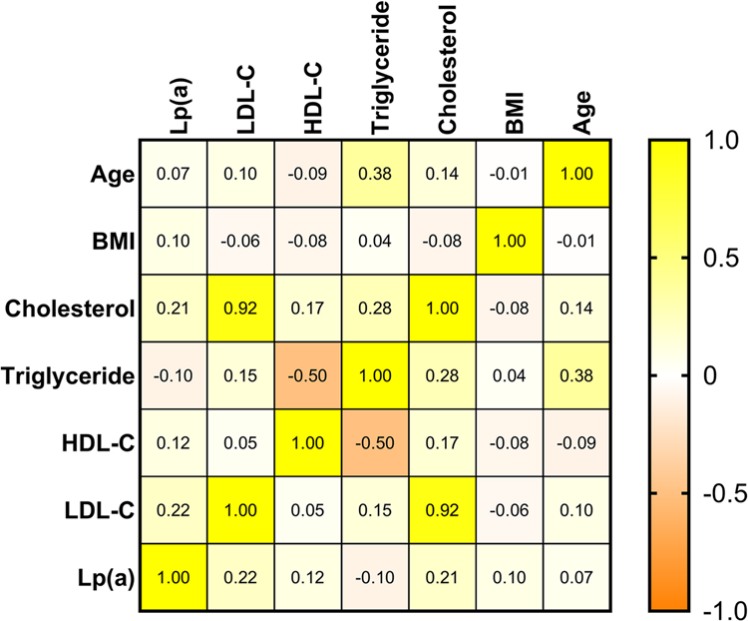
Correlation matrix of Lp(a) levels and other clinical variables. Abbreviations: BMI: body mass index; HDL‐C: high‐density lipoprotein cholesterol; LDL‐C: low‐density lipoprotein cholesterol; Lp(a): lipoprotein(a).

The correlation matrix between Lp(a), lipid profiles, BMI, and age indicates that Lp(a) has no significant correlation with other lipid parameters, BMI, or age. A strong positive correlation was observed between LDL‐C and total cholesterol (*r*_s_ = 0.92), whereas an inverse correlation existed between HDL‐C and triglyceride (*r*_s_ = −0.50). Furthermore, triglyceride were positively correlated with age (*r*_s_ = 0.38). Additional details are presented in Figure 3.

**Figure 4:**
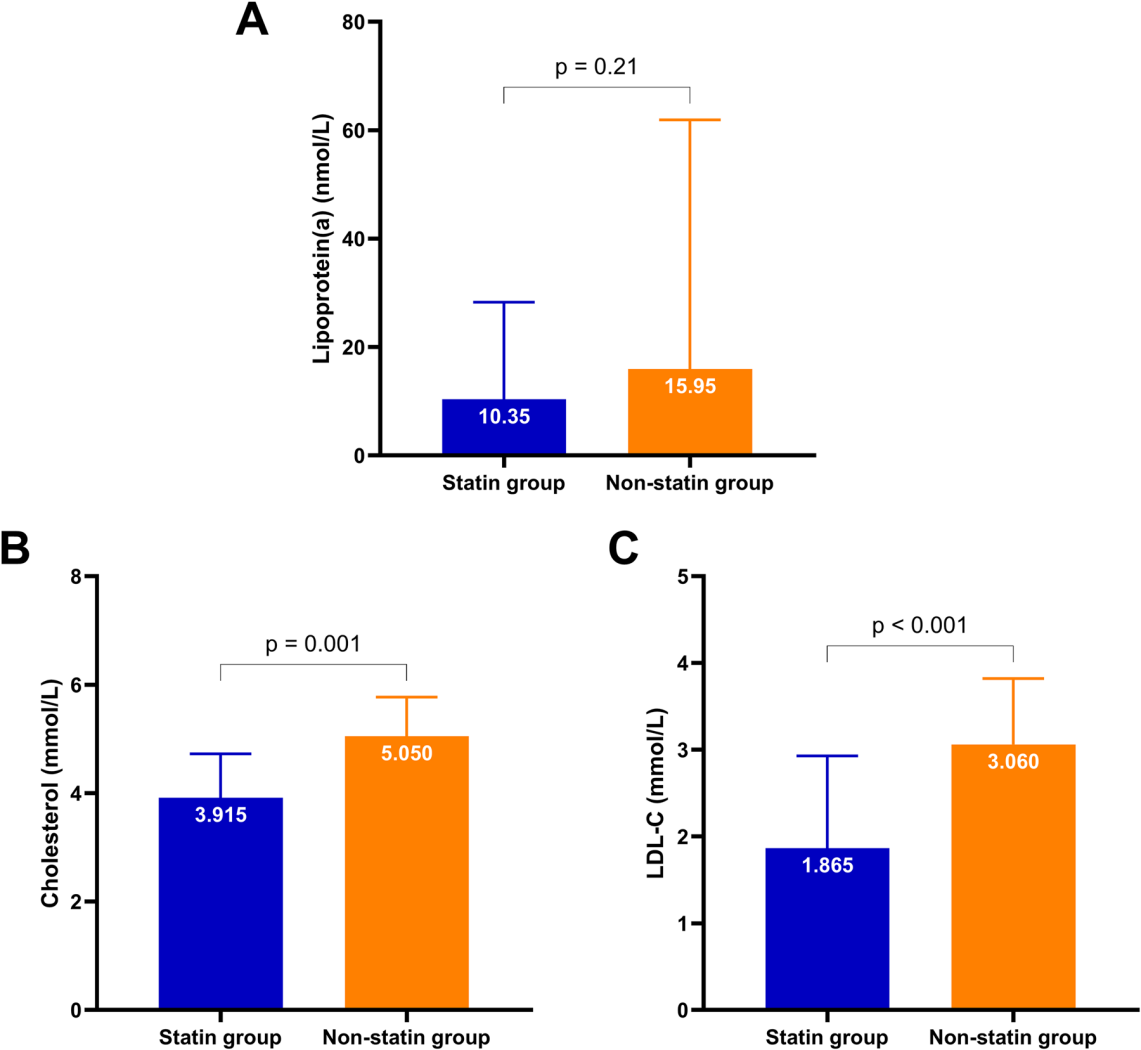
Comparison of lipid profiles between the statin group and the nonstatin group. (A) Lp(a) levels; (B) total cholesterol levels; and (C) LDL‐C levels. Abbreviation: LDL‐C, low‐density lipoprotein cholesterol.

Figure 4 illustrates the differences in lipid profiles between participants who used statins (statin group) and those who did not use statins (nonstatin group). Compared with the statin group, the nonstatin group presented higher total cholesterol (5.050 mmol/L vs. 3.915 mmol/L) and LDL‐C (3.060 mmol/L vs. 1.865 mmol/L) levels. However, there was no significant difference in Lp(a) levels between the two groups.

Finally, a subgroup analysis based on Lp(a) levels revealed that the median age did not differ significantly between the two groups (*p* = 0.488). Additionally, there was no significant difference in sex distribution between the two groups (*p* = 0.328). Other details are presented in Table 2.

**Table 2:** Comparison of characteristics between groups with Lp(a) levels ≥ 125 nmol/L and those with Lp(a) levels < 125 nmol/L.

Characteristics	Lp(a) ≥ 125 nmol/L (*n* = 20)	Lp(a) < 125 nmol/L (*n* = 145)	*p* value
Age (years)	34 [27–46]	33 [26–41]	0.488
Female (%)	15 (75.0)	90 (62.1)	0.328
Hypertension (%)	1 (5.0)	16 (11.0)	0.697
Diabetes (%)	0 (0.0)	4 (2.8)	> 0.99
Smoking (%)	0 (0.0)	5 (3.4)	> 0.99
Alcohol consumption (%)	5 (25.0)	21 (14.5)	0.322
Physical inactivity (%)	3 (15.0)	16 (11.0)	0.707
BMI (kg/m^2^)	23.84 ± 3.42	22.08 ± 2.74	**0.043**
Cholesterol (mmol/L)	5.50 [4.34–5.92]	4.95 [4.27–5.64]	0.351
Triglycerid (mmol/L)	1.42 [1.05–2.48]	1.56 [0.98–2.39]	0.896
HDL‐C (mmol/L)	1.30 [1.14–1.71]	1.36 [1.11–1.55]	0.892
LDL‐C (mmol/L)	3.15 [2.56–4.21]	3.03 [2.47–3.65]	0.454
Non‐HDL‐C(mmol/L)	3.94 [2.97–4.66]	3.48 [2.87–4.28]	0.328

*Note:* The values are presented as the means ± standard deviations, medians [IQRs], or *n* (%) as appropriate.

Abbreviations: BMI, body mass index; HDL‐C, high‐density lipoprotein cholesterol; LDL‐C, low‐density lipoprotein cholesterol; Lp(a), lipoprotein(a).

**Figure 5:**
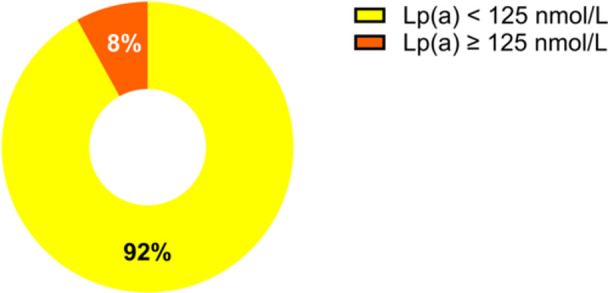
Proportion of patients with and without elevated Lp(a) among those achieving LDL‐C and non‐HDL‐C targets.

Among the subgroup of patients who achieved LDL‐C and non‐HDL‐C targets as per recommendations, 8% still presented elevated Lp(a) levels (≥ 125 nmol/L) (Figure 5). This highlights the persistence of residual cardiovascular risk associated with elevated Lp(a), even in patients with optimal lipid control.

## 4 Discussion

### 4.1 Challenges of Elevated Lp(a) in Healthcare Systems

Our study revealed that Lp(a) was not strongly correlated with cholesterol, triglyceride, LDL‐C, or HDL‐C levels. Additionally, there were no significant differences in age, sex, cholesterol, triglyceride, LDL‐C, or HDL‐C levels between the elevated Lp(a) group and the nonelevated Lp(a) group. Unlike other lipoproteins, Lp(a) levels are genetically determined, stable throughout life, and minimally influenced by environmental factors [21]. While the physiological role of Lp(a) remains unclear, studies suggest its involvement in wound healing and vascular remodeling. Nevertheless, Lp(a) is prone to oxidation, forming pro‐inflammatory and proatherogenic oxidized phospholipids [22]. Lp(a) is considered more atherogenic than LDL is, as it not only contains all the atherogenic components of LDL but also possesses unique thrombotic properties due to apolipoprotein(a) [23]. Apolipoprotein(a) promotes arterial and venous thrombosis through mechanisms such as increased oxidized phospholipid levels, accumulation in vascular walls, and impaired plasminogen activation [23, 24].

A cohort study in the United States revealed that within the first year after an ASCVD diagnosis, patients with Lp(a) levels ≥ 150 nmol/L had higher hospitalization rates, longer hospital stays per admission, and increased cardiovascular events (myocardial infarction, stroke, and coronary revascularization) [25]. Additionally, in patients undergoing percutaneous coronary intervention, those with Lp(a) > 75 nmol/L had significantly higher rates of ischemic recurrence and repeat revascularization than those with Lp(a) ≤ 75 nmol/L [26]. Lp(a) also independently predicts revascularization risk in patients with acute coronary syndrome and diabetes [27]. These findings underscore the significant contribution of elevated Lp(a) levels to the economic and clinical burden associated with ASCVD.

In this investigation, Lp(a) levels were not significantly different in terms of the mean or median values between males and females. Using the American College of Cardiology and American Heart Association recommended threshold of ≥ 125 nmol/L [19], the prevalence of elevated Lp(a) was 12.12%, which was consistent across sexes. Despite being a small pilot study with a limited sample size, the prevalence of elevated Lp(a) among participants aligns with the reported prevalence in Asia, which ranges from 10% to 25% [14, 15, 16]. Our findings indicate that one in ten individuals may have elevated Lp(a) levels. This prevalence underscores the importance for Vietnam's healthcare system to prioritize Lp(a) testing for screening and managing individuals at risk, ultimately providing appropriate solutions to reduce the burden of elevated Lp(a).

### 4.2 Therapeutic Gaps

The analysis revealed that there was no significant difference in Lp(a) levels between the statin group and the nonstatin group, despite statins being the first‐line therapy for dyslipidemia management. This finding aligns with previous reports indicating that while statins are effective at lowering LDL‐C levels, they have a limited effect on Lp(a) [9].

Currently, there are no clinically approved therapies specifically designed to lower Lp(a). Genetic studies suggest that a reduction of at least 130 nmol/L is needed to achieve the same cardiovascular benefits as a 1 mmol/L reduction in LDL‐C [28]. Additionally, the HPS2‐THRIVE trial demonstrated that reducing Lp(a) levels by at least 80 nmol/L (a 40% baseline reduction) can significantly reduce ASCVD‐related cardiovascular events [29].

Given that Lp(a) levels are primarily determined by genetics and are minimally affected by diet or environmental factors [21], emerging therapies such as pelacarsen [30], olpasiran [31], and SLN360 [32] are currently under investigation in clinical trials. Until these therapies become available, strict management of traditional cardiovascular risk factors remains the main strategy to mitigate ASCVD risk in individuals with elevated Lp(a) levels [33].

### 4.3 Prospects for Lp(a) Application in Clinical Practice in Vietnam and Developing Countries

A study conducted in Sweden revealed significantly higher costs for ASCVD patients than for non‐ASCVD patients [34]. Controlling ASCVD through the management of cardiovascular risk factors can reduce disease progression and mortality, thereby alleviating direct and indirect healthcare costs [34, 35]. Lp(a) levels are used in cardiovascular risk stratification, where higher Lp(a) levels necessitate more stringent LDL‐C reduction targets. This enhances the control of traditional cardiovascular risk factors, especially in the absence of effective Lp(a)‐lowering therapies [36]. Both the European Society of Cardiology, the European Atherosclerosis Society, the American College of Cardiology, and the American Heart Association recommend measuring Lp(a) at least once in a lifetime, particularly in high‐risk individuals or those with a family history of early‐onset cardiovascular disease [11, 19].

Subgroup analysis of patients who achieved LDL‐C and non‐HDL‐C treatment targets revealed that 8% still had elevated Lp(a) levels. This finding is concerning, as Lp(a) represents a “hidden iceberg” of cardiovascular risk that could pose threats at any time. This raises a critical question: Should Lp(a) testing be conducted more frequently and expanded to include individuals who have already achieved optimal control of traditional lipid parameters?

In summary, Lp(a) testing allows for the early identification of high‐risk patients and timely lipid‐lowering interventions, ultimately reducing the economic burden associated with ASCVD. On the basis of our promising preliminary results, despite the small sample size, it is crucial to take action by conducting further studies and organizing conferences to increase knowledge about Lp(a). These efforts will help expand the application of Lp(a) testing in clinical practice in Vietnam and other developing countries.

### 4.4 Limitations

This study has several limitations that should be acknowledged. First, as a cross‐sectional design, it does not allow for the assessment of the long‐term impact of elevated Lp(a) on mortality, nor does it explore the underlying pathophysiological mechanisms. Second, being a pilot study conducted at a single event with a relatively small sample size, the single‐center design may limit external validity and introduce selection bias.

Third, the influence of lifestyle factors such as diet, work intensity, and the mental health status of physicians was not controlled for, which may have affected the observed Lp(a) levels. In addition, factors such as sex hormones that are known to influence Lp(a) were not assessed. Fourth, since the study population consisted exclusively of physicians, a specific and health‐conscious group, the generalizability of the findings to the broader Vietnamese population is limited.

Finally, owing to the lack of a nationwide electronic health record system in Vietnam, data on comorbidities and medication use were collected through self‐report questionnaires, which may be subject to recall bias.

### 4.5 Future Research Directions

This pilot study lays the foundation for further research on Lp(a) in Vietnam. Larger, multicenter studies are needed to validate our findings and explore the clinical utility of Lp(a) in risk assessment and management. Collaborative efforts through research and conferences are essential to enhance knowledge, expand the use of Lp(a) testing in clinical practice, and address its implications in Vietnam and other developing countries.

## 5 Conclusion

At the 2nd Vietnam Atherosclerosis Society Congress, elevated Lp(a) levels were observed in a number of cardiologists without prior cardiovascular disease. Interestingly, some of these individuals also had lipid profiles that were well‐controlled according to current guidelines. These findings suggest that Lp(a) may provide additional insight beyond traditional lipid markers, highlighting the need to consider Lp(a) screening as part of a broader strategy to improve cardiovascular risk assessment. Further research with larger and more diverse populations is needed to clarify its clinical value in the Vietnamese context.

## Author Contributions

**Thanh Huong Truong:** writing – original draft, writing – review and editing, methodology, investigation, formal analysis, data curation, conceptualization. **Tien Anh Hoang:** writing – original draft, writing – review and editing, methodology, investigation, formal analysis, conceptualization. **Van Sy Hoang:** writing – original draft, writing – review and editing, conceptualization. **Ngoc Quang Nguyen:** conceptualization, writing – review and editing. **Hoa Tran:** writing – original draft, writing – review and editing, validation, supervision, methodology, investigation, funding acquisition, conceptualization, project administration, resources. **Duc Chinh Nguyen:** investigation, conceptualization, writing – review and editing. **Huu Duc Nguyen:** investigation, conceptualization, writing – review and editing. **Hai Nguyen Ngoc Dang:** writing – original draft, writing – review and editing, visualization, formal analysis, data curation, investigation, conceptualization. **Thang Viet Luong:** conceptualization, investigation, writing – original draft, writing – review and editing. **Doan Loi Do:** conceptualization, investigation, writing – review and editing. **Mai Ngoc Thi Nguyen:** writing – review and editing, conceptualization. **Hong An Le:** conceptualization, writing – review and editing. **Thanh Tung Le:** conceptualization, writing – review and editing. **Ngoc Thanh Kim:** conceptualization, writing – review and editing, investigation. **Quang Binh Truong:** conceptualization, methodology, investigation, supervision, writing – review and editing, validation.

## Ethics Statement

Our research was approved by the Vietnam Atherosclerosis Society (approval number: 01/VAS‐NCKH). The research was conducted following the guidelines stipulated in the Helsinki Declaration (2013). In addition, for investigations involving human subjects, informed consent was obtained from the participants involved.

## Conflicts of Interest

The authors declare no conflicts of interest.

## Data Availability

The data that support the findings of this study are available from the corresponding author upon reasonable request.
